# Single-Chain Variable Fragments Targeting Plasma Exosome Amyloid-β Peptide Subtypes for Diagnosis of Alzheimer’s Disease

**DOI:** 10.3390/ijms272110006

**Published:** 2026-11-08

**Authors:** Xiaojin Zhou, Zan Wang, Hui Jiang

**Affiliations:** State Key Laboratory of Digital Medical Engineering, School of Biological Science and Medical Engineering, Southeast University, Nanjing 211189, China

**Keywords:** Alzheimer’s disease, single-chain variable fragment, β-amyloid peptide, plasma exosome, chemiluminescence immunoassay, diagnostic biomarker

## Abstract

The diagnostic value of the Aβ1-42/Aβ1-40 ratio in peripheral blood as a biomarker for Alzheimer’s disease (AD) remains a subject of considerable interest. However, this ratio in plasma exosomes is still underestimated. To investigate the potential clinical performances, this study has established a chemiluminescence immunoassay (CLIA) for the specific detection of Aβ1-42 and Aβ1-40 in plasma exosomes using single-chain variable fragments (scFv). Plasma samples from 20 AD patients and 20 non-AD individuals were detected by the established scFv-based CLIA, with the traditional monoclonal antibody (mAb)-based CLIA as the reference method. The results demonstrated that the plasma exosome Aβ1-42/Aβ1-40 ratio in AD patients (0.70 ± 0.35) was significantly lower than that in the non-AD control group (1.82 ± 0.79, *p* < 0.001). In comparison with the mAb-based assay, the scFv-based CLIA exhibited superior analytical performance, with a lower limit of detection (1 pg/mL vs. 50 pg/mL) and higher specificity (cross-reactivity rate < 5%). When the Aβ1-42/Aβ1-40 ratio of 1.075 was set as the cutoff value, the scFv-based assay achieved a diagnostic sensitivity of 90.0%, which was numerically higher than that of the mAb-based assay (85.0%), with comparable specificity (90.0% vs. 90.0%). Generally, this study has developed a highly sensitive and specific scFv-based method for the detection of plasma exosome Aβ subtypes, which supports that the plasma exosome Aβ1-42/Aβ1-40 ratio is a potential non-invasive biomarker for differentiating clinically diagnosed AD from non-AD controls in this pilot cohort.

## 1. Introduction

Alzheimer’s disease (AD) is the most prevalent neurodegenerative dementia in the elderly population, pathologically characterized by diffuse cerebral cortical atrophy, intracellular neurofibrillary tangles (NFTs) and extracellular senile plaques (SPs) formed by β-amyloid (Aβ) aggregation, and clinically manifested as progressive cognitive impairment and memory deterioration as the core symptoms [1,2]. AD is an irreversible progressive neurodegenerative disease accounting for 60–80% of all dementia cases worldwide [1]. According to the *2023 World Alzheimer Report* [1], the global number of dementia patients is approximately 55 million, and this figure is projected to rise to 139 million by 2050, with 60–70% of these cases attributed to AD. In China, epidemiological data show that there are about 15.07 million dementia patients aged 60 years and above, accounting for 27% of the global dementia population, among which 9.83 million are diagnosed with AD, imposing a heavy burden on families and the whole society [2].

Although the pathogenesis of AD has not been fully elucidated, the abnormal deposition of Aβ plaques in the brain is a well-recognized core pathological hallmark [3]. Aβ oligomers are the primary neurotoxic substances driving neuronal damage and cognitive decline, which is the core theoretical basis of the amyloid cascade hypothesis [4]. Current clinical diagnosis of AD mainly relies on neuroimaging examinations and neuropsychological cognitive function assessments, which suffer from the limitations of delayed diagnosis and insufficient specificity [5,6]. Traditional Aβ detection methods, including cerebrospinal fluid (CSF) collection via lumbar puncture and Aβ positron emission tomography (PET) imaging, are either invasive or costly, making them unsuitable for large-scale population screening and early AD diagnosis [6,7]. Dried blood spot sampling provides a new minimally invasive sampling mode for large-scale AD population screening, but its detection stability still needs further clinical verification [8]. In recent years, blood-based Aβ detection has become a research hotspot in the field of AD diagnosis due to its advantages of convenience, rapidity and low cost [9,10]. Existing blood-based Aβ detection technologies are mainly divided into immunoassay-based methods (e.g., single molecule enzyme-linked immunosorbent assay [Simoa], or electrochemiluminescence immunoassay) and mass spectrometry (MS)-based approaches (e.g., immunoprecipitation-mass spectrometry [IP-MS], or liquid chromatography-tandem mass spectrometry [LC-MS/MS]) [9,10]. Simoa relies on single-molecule microarray technology to realize ultra-low concentration Aβ quantification in plasma and can detect Aβ oligomer species that mediate neurotoxicity [7,11]; IP-MS-derived assays such as PrecivityAD™ and its upgraded version PrecivityAD™2 have been clinically validated for the identification of cerebral amyloid status by combining plasma Aβ and p-tau217 indicators [12,13,14]. The pretreatment optimization scheme of IP-MS/LC-MS/MS for plasma exosomal Aβ has also been continuously reported to improve quantitative accuracy [12,15]. Simoa and most IP-MS/LC-MS/MS plasma Aβ assays primarily measure plasma total or free Aβ, whereas exosome-based assays require prior EV isolation. However, these methods still have inherent drawbacks, including insufficient sensitivity and specificity, high operational costs, stringent sample pretreatment requirements, limited ability to detect Aβ aggregates, poor pre-analytical stability of plasma samples, and poor generalizability across different populations [8,15,16,17,18,19,20,21,22], which restrict their clinical application. Electrochemical biosensors based on CRISPR technology have also been tried for simultaneous detection of Aβ40 and Aβ42, but they cannot be applied to exosomal Aβ detection in peripheral blood at present [23].

Exosomal Aβ refers to Aβ associated with plasma exosomes, including Aβ on the EV membrane and within the EV lumen. Normally, exosomes were isolated and lysed, so the assay measures total exosome-associated Aβ. Free plasma Aβ was removed during exosome isolation; however, co-isolation of free Aβ cannot be excluded without further validation [24,25,26,27]. Blood extracellular vesicles carrying synaptic proteins and NCAM-labeled exosomal Aβ42 can realize early identification of subjective cognitive decline and preclinical AD [28,29,30,31]. In the sandwich CLIA, anti-Aβ1-16 is coated on magnetic beads as capture, and ALP-conjugated anti-Aβ1-40 or anti-Aβ1-42 is used as detection. After exosome isolation and lysis, the lysate is added to the capture beads. The assay therefore measures Aβ released from exosomes rather than free plasma Aβ [18,19]. In addition, the meta-analysis and clinical cohort studies have confirmed that the plasma exosome Aβ1-42/Aβ1-40 ratio has high diagnostic efficiency for AD pathological changes and is expected to become a core auxiliary diagnostic indicator [8,12,13,14].

**Scheme 1:**
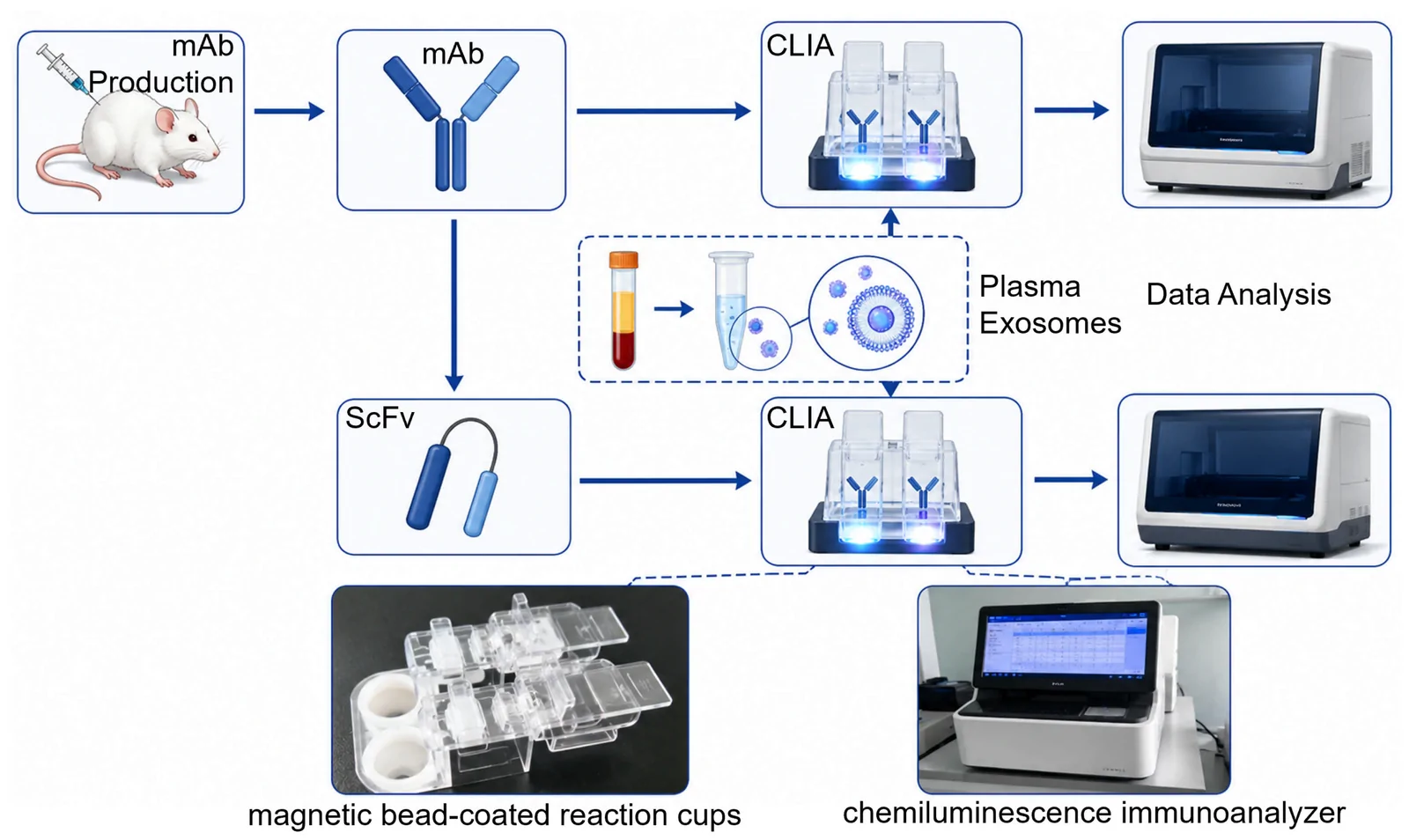
The design of mAbs and scFv for CLIA of Aβ1-40 and Aβ1-42 in plasma exosomes.

To overcome these disadvantages, we proposed an alternative detection strategy based on single-chain variable fragments (scFv). ScFv are engineered antibody fragments composed of the variable heavy chain (VH) and variable light chain (VL) of monoclonal antibody (mAb) linked by a flexible peptide linker. They possess the advantages of small molecular weight, high stability, low immunogenicity and cost-effective recombinant production, while retaining the specific antigen-binding activity of the parent mAb [32,33,34,35]. Recombinant scFv targeting Aβ epitopes can be constructed by screening specific antibody libraries, and scFv modified by surface engineering can significantly improve the capture capacity of exosome-bound Aβ aggregates [35,36,37]. These characteristics make scFv ideal candidates for the development of improved in vitro diagnostic assays for AD [38,39]. In this study, we prepared scFv specifically targeting Aβ1-16, Aβ1-40 and Aβ1-42, established an scFv-based double-antibody sandwich chemiluminescence immunoassay (CLIA) for the detection of Aβ1-40 and Aβ1-42 in plasma exosomes, and compared its detection performance with the traditional mAb-based CLIA (Scheme 1). We also explored the diagnostic value of the plasma exosome Aβ1-42/Aβ1-40 ratio as an AD biomarker, aiming to provide a new technical method for the early clinical diagnosis of AD.

## 2. Results

### 2.1. Participants

Participants were divided into two groups: healthy controls (HC) and patients with clinically diagnosed Alzheimer’s disease (AD) (Table 1). Healthy controls were retrospectively identified from a routine health check-up database. Eligible HC subjects had no self-reported cognitive impairment and no prior diagnosis of dementia or neurodegenerative disease. Standardized cognitive scale testing (MMSE or MoCA) was not available for this retrospective health-screening cohort. AD patients were recruited from the memory clinic and diagnosed according to the NIA-AA 2018, with comprehensive clinical and cognitive assessment.

**Table 1:** Baseline characteristics of the HC and AD groups.

Characteristic	HC (*n* = 20)	AD (*n* = 20)	*p* Value
Age (years), mean ± SD	61.15 ± 8.50	59.95 ± 8.41	0.66
Sex, male/female	10/10	10/10	1.00
Education (years), mean ± SD	9.95 ± 4.29	9.20 ± 4.12	0.58

### 2.2. Preparation and Identification of Anti-Aβ mAb

**Figure 1:**
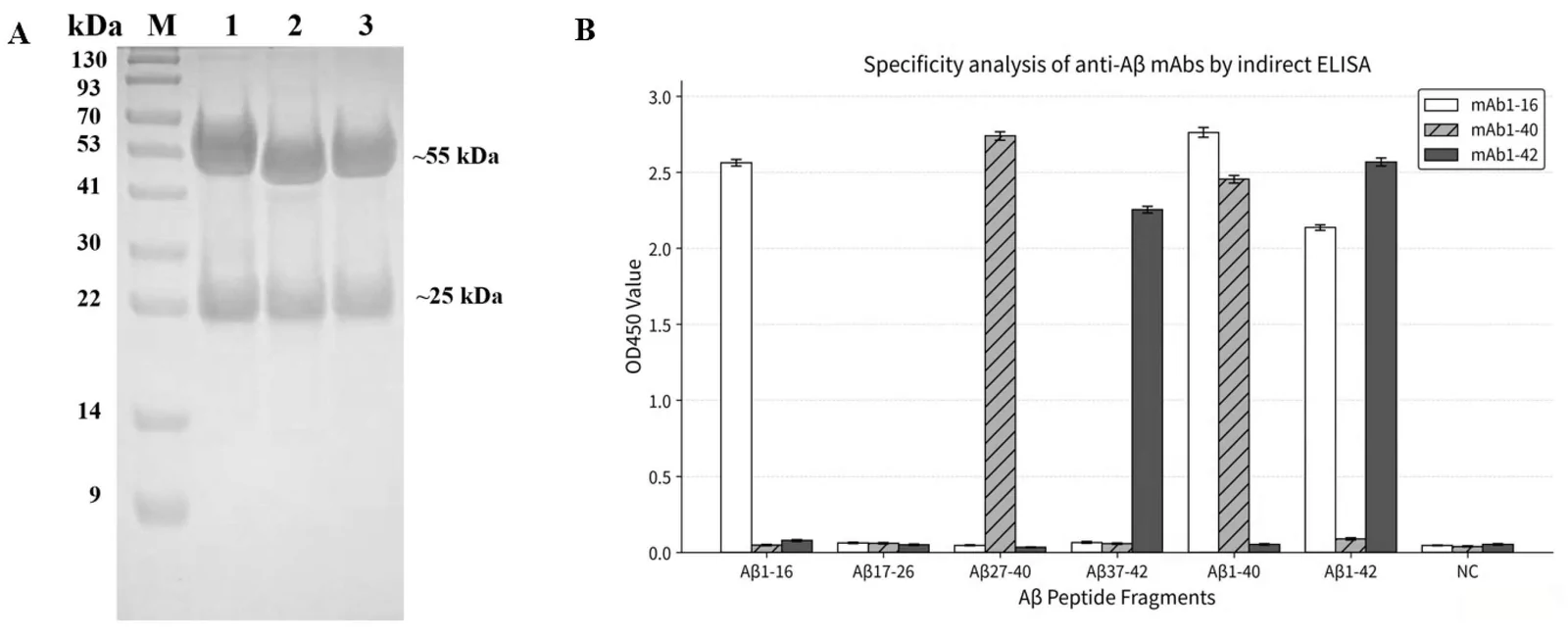
(**A**) SDS-PAGE analysis of purified anti-Aβ mAbs. Lane M: Protein Marker; Lane 1: Purified mAb1-16; Lane 2: Purified mAb1-40; Lane 3: Purified mAb1-42. (**B**) Specificity analysis of anti-Aβ mAb by indirect ELISA. The histogram shows the OD_450_ values of three mAbs binding to different Aβ peptide fragments. All mAbs show no obvious binding to non-target fragments (dashed line, OD_450_ < 0.1). Data are presented as mean ± S.D. (n = 3).

Three hybridoma cell lines specifically secreting anti-Aβ1-16, Aβ1-40 and Aβ1-42 mAb were successfully obtained by cell fusion and screening, and the corresponding mAbs were purified by protein A affinity chromatography. Figure 1A showed the SDS-PAGE results of the purified mAb, with two specific bands (~50 kDa for heavy chain, ~25 kDa for light chain) and no obvious impurity bands, indicating high purity of the prepared antibodies.

Indirect ELISA results for epitope mapping and specificity analysis (Figure 1B, Table S1) indicated that mAb1-16 specifically bound to Aβ1-16, and also cross-bound to the full-length Aβ1-40 and Aβ1-42 peptides (OD_450_ of 2.0 as threshold). Similarly, mAb1-40 specifically bound to the Aβ27-40 epitope and full-length Aβ1-40, and mAb1-42 specifically bound to the Aβ37-42 epitope and full-length Aβ1-42. All three mAbs showed almost no binding to non-target Aβ fragments (dashed line, OD_450_ of 0.1 as threshold,), and the OD_450_ values of the negative control were all less than 0.07, demonstrating high subtype specificity of the prepared mAb.

### 2.3. Preparation and Characterization of Anti-Aβ scFv

**Figure 2:**
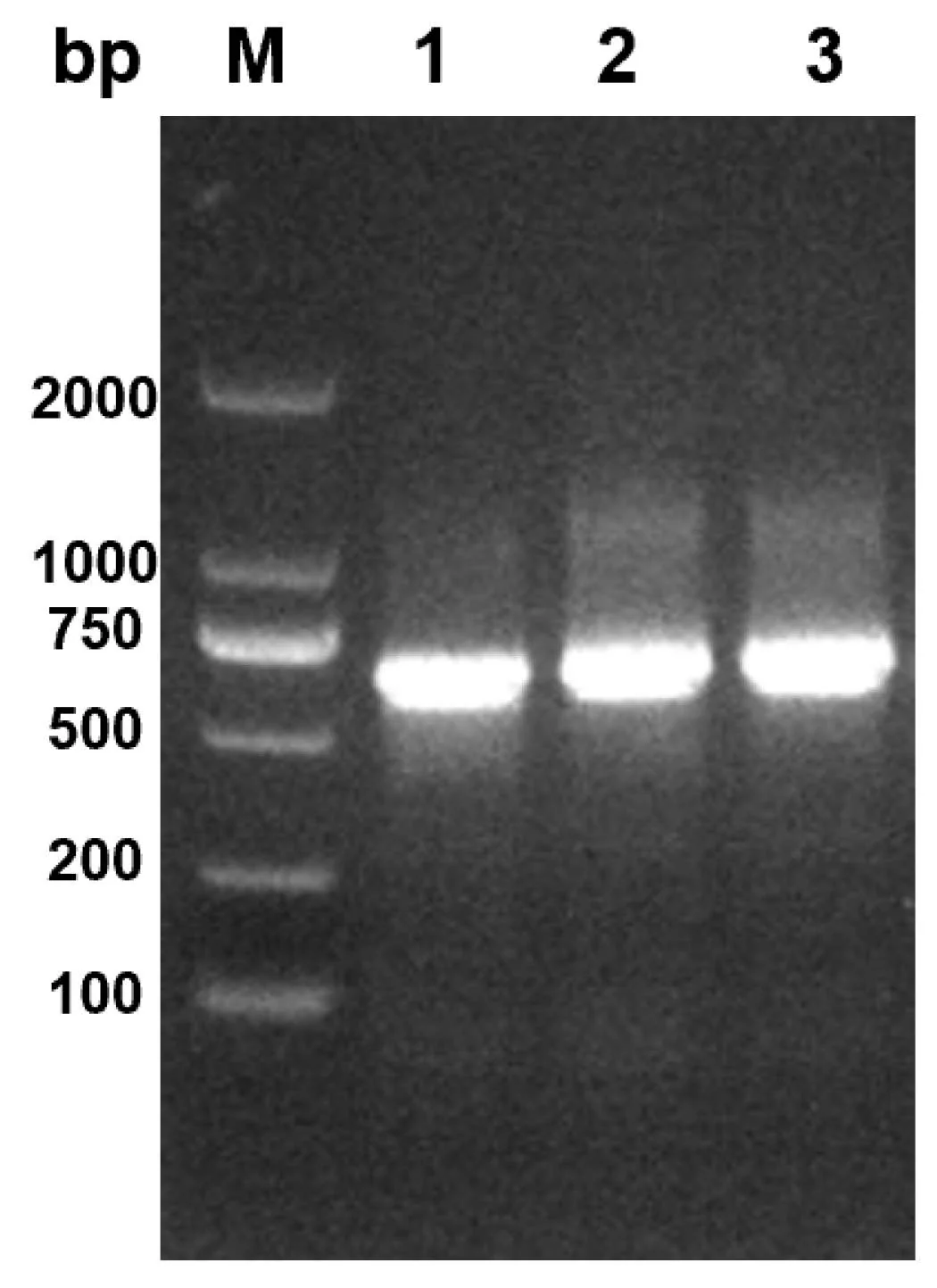
PCR verification of pET-28a-scFv recombinant expression vectors. Lane M: DNA Marker; Lane 1: pET-28a-scFv1-16; Lane 2: pET-28a-scFv1-40; Lane 3: pET-28a-scFv1-42.

Recombinant expression vectors pET-28a-scFv1-16, pET-28a-scFv1-40, and pET-28a-scFv1-42 were successfully constructed. Colony PCR verification (Figure 2) showed a specific band (735–738 bp) for all three vectors, which was consistent with the expected size of the scFv gene insert (729–732 bp; VH—linker—VL) plus the restriction-site/flanking sequences. Sanger sequencing results (Supporting Information) confirmed no base mutations, deletions or insertions in the scFv gene sequences.

**Figure 3:**
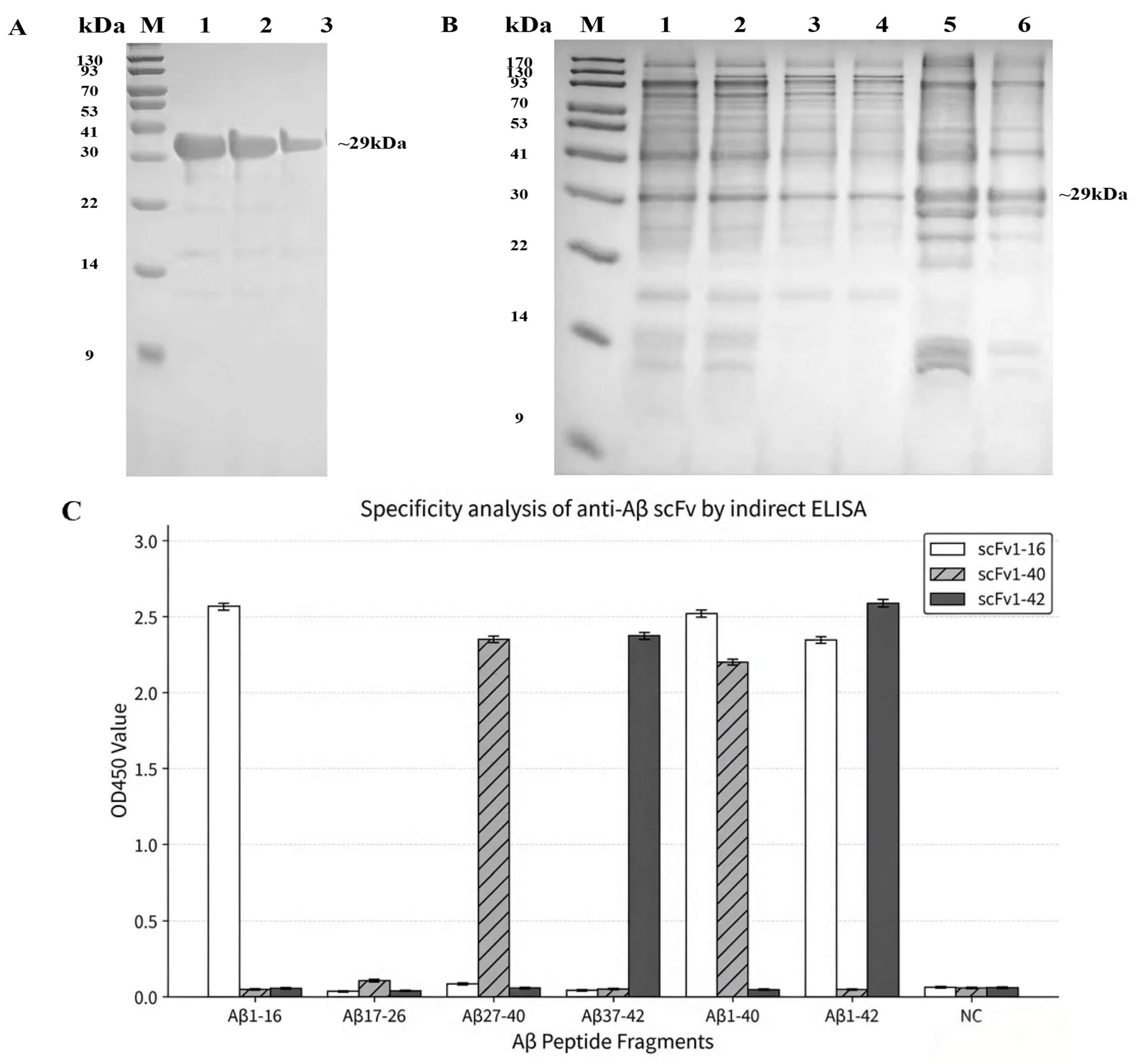
(**A**,**B**) SDS-PAGE analysis of induced expression and purification of anti-Aβ scFv. (**A**) Induced expression of scFv. M: Protein Marker; Lane 1: scFv1-16 (before induction); Lane 2: scFv1-16 (after IPTG induction); Lane 3: scFv1-40 (before IPTG induction); Lane 4: scFv1-40 (after IPTG induction); Lane 5 scFv1-42 (before IPTG induction); Lane 6 scFv1-42 (after IPTG induction). (**B**) Purification of scFv. Lane M: Protein Marker; Lane 1: Purified scFv1-16; Lane 2: Purified scFv1-40; Lane 3: Purified scFv1-42. (**C**) Specificity analysis of anti-Aβ scFv by indirect ELISA. The histogram shows the OD_450_ values of three scFv binding to different Aβ peptide fragments. The cross-reactivity with non-target fragments is extremely low (dashed line, OD_450_ < 0.12). Data are presented as mean ± S.D. (n = 3).

SDS-PAGE results showed that a new specific protein band of approximately 29 kDa appeared in *E. coli* BL21(DE3) lysate after IPTG induction (Figure 3A), consistent with the predicted molecular weight of recombinant scFv with a 6 × His tag. After Ni-IDA affinity chromatography purification, a single specific band at 29 kDa was observed (Figure 3B), with protein purity exceeding 95% by gray value analysis, indicating successful prokaryotic expression and high-purity purification of scFv.

Indirect ELISA confirmed that the scFv retained epitope-binding reactivity comparable to the parental mAbs (Table S2, Figure 3C). Generally, scFv1-16 specifically bound to Aβ1-16, Aβ1-40 and Aβ1-42 (OD_450_ > 2.0), while scFv1-40 specifically bound to Aβ27-40 and Aβ1-40, and scFv1-42 specifically bound to Aβ37-42 and Aβ1-42. The cross-reactivity rate of scFv with non-target Aβ fragments was less than 5%, confirming high specificity of the prepared scFv. Cross-reactivity was calculated as: cross-reactivity (%) = (OD_450non-target_ − OD_450blank_)/(OD_450target_ − OD_450blank_) × 100%.

### 2.4. Analytical Performance of mAb-Based and scFv-Based CLIA

**Figure 4:**
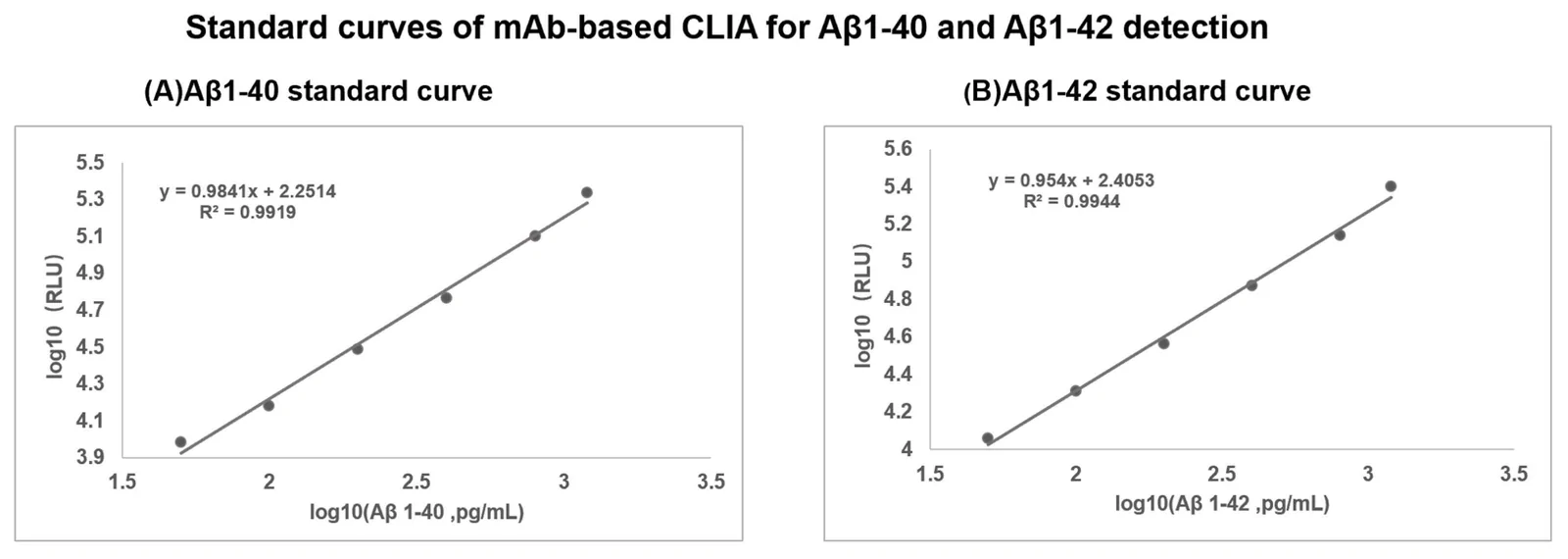
Standard curves of mAb-based CLIA for the detection of (**A**) Aβ1-40 and (**B**) Aβ1-42.

**Figure 5:**
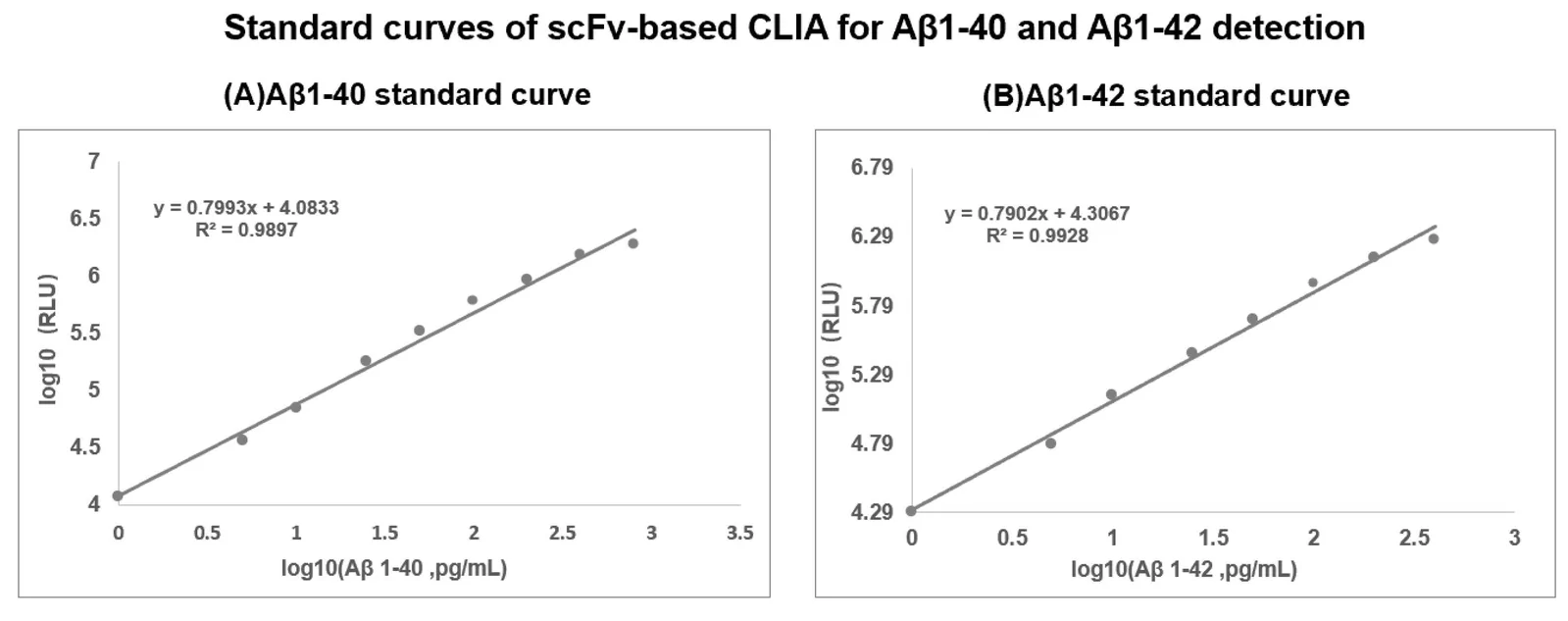
Standard curves of scFv-based CLIA for the detection of (**A**) Aβ1-40 and (**B**) Aβ1-42.

Based on above screened mAbs and scFv, the standard curves for Aβ1-40 and Aβ1-42 were established by mAb-based and scFv-based CLIA, respectively (Table S3). By using mAb-based CLIA (Figure 4), the linear detection ranges are 50–1200 pg/mL for both Aβ1-40 and Aβ1-42, with regression equations of log10(RLU) = 0.9841 × log10(C) + 2.2514 (R^2^ = 0.9919) for Aβ1-40 and log10(RLU) = 0.9540 × log10(C) + 2.4053 (R^2^ = 0.9944) for Aβ1-42, and LODs of 50 pg/mL for both analytes. In contrast, by using scFv-based CLIA (Figure 5), the linear detection ranges are 1–800 pg/mL for Aβ1-40 and 1–400 pg/mL for Aβ1-42, with regression equations of log10(RLU) = 0.7993 × log10(C) + 4.0833 (R^2^ = 0.9897) and log10(RLU) = 0.7902 × log10(C) + 4.3067 (R^2^ = 0.9928), respectively, and LODs of 1 pg/mL for both analytes.

### 2.5. Detection of Plasma Exosome Aβ1-40 and Aβ1-42 in AD and Non-AD Groups

**Figure 6:**
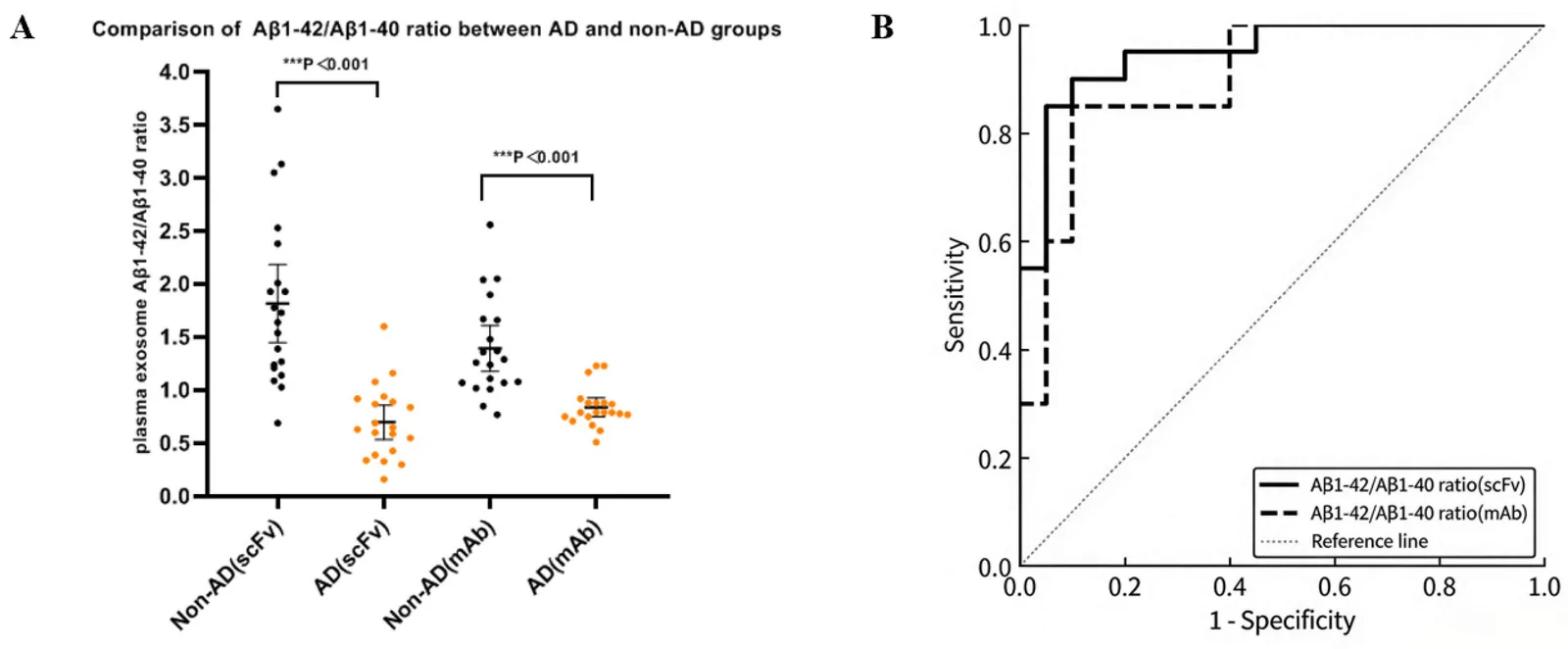
(**A**) Comparison of plasma exosome Aβ1-42/Aβ1-40 ratios between AD and non-AD groups (detected by scFv/mAb-based CLIA); (**B**) ROC curves of plasma exosome Aβ1-42/Aβ1-40 ratio (scFv/mAb-based CLIA).

The scFv-based and mAb-based CLIA were used to detect the concentrations of Aβ1-40 and Aβ1-42 in the plasma exosome lysates of 20 AD patients (meeting the NIA-AA 2018 AD diagnostic criteria) and 20 age- and gender-matched non-AD individuals (Table 2). The non-AD group showed a high SD (0.79), indicating substantial heterogeneity. This may affect cutoff stability. Figure 6A shows the comparison of the plasma exosome Aβ1-42/Aβ1-40 ratio between the two groups detected by scFv/mAb-based assay. For the scFv assay, the ratio in the AD group (0.70 ± 0.35) was significantly lower than that in the non-AD control group (1.82 ± 0.79) (t = −5.838, *p* < 0.001), indicating a significant difference between the two groups. Similarly, based on the mAb assay, the ratio in the AD group (0.84 ± 0.19) was lower than that in the non-AD control group (1.39 ± 0.46) (t = −4.992, *p* < 0.001), with a significant difference between the two groups.

ROC curve analysis was performed with the plasma exosome Aβ1-42/Aβ1-40 ratio (detected by scFv/mAb-based assay) as the diagnostic index (Figure 6B). The scFv-based assay yielded an AUC of 0.948 (95% CI: 0.873–0.998, *p* < 0.001), while the mAb-based assay yielded an AUC of 0.900 (95% CI: 0.800–1.000, *p* < 0.001). The difference between the two AUCs was not statistically significant (DeLong test, *p* = 0.780).

Based on the optimal cutoff value of 1.075 for the scFv-based assay and 0.924 for the mAb-based assay, the diagnostic performance of the two assays was compared (Table 3 and Table S4), and the intra- and inter-assay CVs were investigated (Table S5). The scFv-based assay achieved a diagnostic sensitivity of 90.0% (18/20) and specificity of 90.0% (18/20) for AD, with an overall accuracy of 90.0%; while the mAb-based assay had a sensitivity of 85.0% (17/20), specificity of 90.0% (18/20) and accuracy of 87.5%. In this pilot cohort, the scFv-based assay showed a numerically higher sensitivity than the mAb-based assay, but the differences between the two assays were not statistically significant (McNemar test, *p* = 0.655; DeLong test for paired AUCs, *p* = 0.780).

## 3. Discussion

Alzheimer’s disease is a progressive neurodegenerative disease with a long preclinical stage, and early diagnosis and intervention are the key strategies to improve the prognosis of AD patients [5]. The lack of sensitive, specific and non-invasive diagnostic biomarkers and detection methods is the main bottleneck restricting the early diagnosis and large-scale screening of AD. In this study, we successfully prepared scFv specifically targeting different Aβ peptide subtypes, established a highly sensitive scFv-based CLIA for the detection of Aβ1-40 and Aβ1-42 in plasma exosomes, and confirmed that the plasma exosome Aβ1-42/Aβ1-40 ratio is a potential non-invasive biomarker for AD diagnosis, which provides a technical approach for further evaluation in AD versus non-AD differentiation.

Exosomes are small extracellular vesicles with a diameter of 30–150 nm that can transport bioactive molecules such as proteins, nucleic acids and lipids between cells [18]. As exosomes in plasma can cross the blood–brain barrier (BBB) through passive diffusion and active transport, and their membrane structure can protect the internal biomolecules from degradation by proteases in the blood, they have become an ideal carrier for the detection of blood-based biomarkers for neurological diseases including AD [18,19]. Aβ peptides are important components of exosome proteins secreted by neural cells, and the imbalance of Aβ subtype expression and aggregation in plasma exosomes is closely associated with the pathological progression of AD. In this study, we found that the plasma exosome Aβ1-42/Aβ1-40 ratio in AD patients was significantly lower than that in non-AD individuals (*p* < 0.001), and the AUC of the ROC curve was 0.948 (95% CI: 0.873–0.998) for the scFv-based assay (0.900, 95% CI: 0.800–1.000 for the mAb-based assay), indicating that the plasma exosome Aβ1-42/Aβ1-40 ratio has good diagnostic value for AD. This result is consistent with the findings of Shaw et al. [11], who reported that the blood Aβ1-42/Aβ1-40 ratio had a high diagnostic value for AD (AUC = 0.89). The underlying mechanism may be that the abnormal aggregation of Aβ1-42 (the more hydrophobic and fibrillogenic subtype) in the brain of AD patients leads to changes in Aβ1-42 secretion and exosome packaging by neural cells, which in turn results in the reduction in the Aβ1-42/Aβ1-40 ratio in plasma exosomes of AD patients [8]. In addition, the Aβ1-42/Aβ1-40 ratio can eliminate the individual differences in total Aβ content, making it a more stable and reliable biomarker than single Aβ subtype [11].

The development of highly sensitive and specific detection methods is the key to the clinical translation of blood-based Aβ biomarkers. Traditional mAb-based immunoassays have inherent limitations in the detection of low-abundance Aβ peptides in plasma, such as low sensitivity and poor specificity [12,15]. The main reasons may include the large molecular weight of mAbs (theoretical ~150 kDa, i.e., ~50 kDa for heavy chain, ~25 kDa for light chain, see Figure 1A), which leads to obvious steric hindrance in the immune reaction. Moreover, the easy denaturation of mAbs in the detection process reduces the antigen-binding efficiency. As genetically engineered antibody fragments, scFv retain the specific antigen-binding activity of the parent mAbs and have the advantages of small molecular weight (~29 kDa), high stability, low immunogenicity and easy recombinant expression [17]. In this study, we constructed scFv by linking the VH and VL region of anti-Aβ mAb with a (Gly4Ser)3 flexible peptide linker and successfully expressed and purified the recombinant scFv in *E. coli* BL21(DE3) cells. SDS-PAGE and ELISA results showed that the prepared scFv had high purity (>95%) and specificity, with a cross-reactivity rate of less than 5% with non-target Aβ fragments, which laid a solid foundation for the development of scFv-based Aβ detection reagents.

We further established an scFv-based double-antibody sandwich CLIA for the detection of Aβ1-40 and Aβ1-42 and systematically compared its analytical performance with traditional mAb-based CLIA. The results showed that the scFv-based CLIA had a significantly lower LOD (1 pg/mL) than the mAb-based CLIA (50 pg/mL), and a wider linear detection range at low concentrations. Both the mAb- and scFv-based standard curves were established under identical coating conditions (0.25 mg/mL capture reagent, mass-based concentration). However, the actual surface-bound molar density was not quantified in this study. A possible explanation for the lower LOD of the scFv-based assay is the higher molar density of the smaller scFv molecule at equal mass concentration, which reduces the steric hindrance effect in the immune reaction and enables more efficient binding to the antigenic epitopes of Aβ peptides (especially the low-abundance Aβ in plasma exosomes). This remains a tentative explanation rather than an established mechanism [13]. In addition, the scFv expressed in prokaryotic cells has a more stable structure, which can maintain high antigen-binding activity in the chemiluminescence reaction system, further improving the detection sensitivity [17]. In the detection of clinical plasma exosome samples, the scFv-based assay achieved a numerically higher diagnostic sensitivity (90.0%) than the mAb-based assay (85.0%), with comparable specificity (90.0% vs. 90.0%) and overall accuracy (90.0% vs. 87.5%). These differences were not statistically significant (McNemar test, *p* = 0.655). The scFv-based CLIA may therefore be a promising alternative for the quantitative detection of low-abundance Aβ peptides in plasma exosomes, although further validation in independent cohorts is required.

Several limitations need to be addressed in future research. In particular, the lack of independent validation, the potential co-isolation of non-exosomal Aβ, and the unquantified molar-equivalent reagent loading in the assay comparison should be regarded as methodological limitations of the present study rather than established analytical advantages. First, the sample size of this study is relatively small (20 AD patients and 20 non-AD individuals), and the study population is a single-center cohort, which may lead to selection bias. The results need to be verified by a large sample of multi-center clinical samples including different age groups, disease stages (preclinical AD, mild cognitive impairment, moderate-severe AD) and ethnic populations. Second, this study only investigated the diagnostic value of the plasma exosome Aβ1-42/Aβ1-40 ratio, and the combination of this ratio with other AD core biomarkers, e.g., phosphorylated tau [p-tau181, p-tau217], neurofilament light chain [NfL], Refs. [9,14] may further improve the diagnostic accuracy of AD, especially for the differential diagnosis of AD and other types of dementia (e.g., vascular dementia, frontotemporal dementia). Third, the scFv in this study was expressed in a prokaryotic expression system (E. coli), which lacks post-translational modifications (e.g., glycosylation) and may affect its biological activity and antigen-binding affinity to a certain extent [10]. Eukaryotic expression systems (e.g., Pichia pastoris, mammalian cells) can be used to express scFv in future studies to further optimize its performance. In addition, the stability and reproducibility of the scFv-based CLIA need to be further verified by long-term storage tests and multi-center clinical trials, and the detection process can be further optimized to improve the detection efficiency and reduce the detection cost, which is conducive to its clinical popularization and application. Fourth, all AD patients were diagnosed according to the NIA-AA 2018 criteria with comprehensive clinical and cognitive assessments, but amyloid PET or CSF biomarker confirmation was not available, and standardized cognitive assessment scales were not administered to the non-AD control group. Moreover, total plasma Aβ was not measured in this study, and a direct comparison between exosome-associated Aβ and total plasma Aβ would require an independent, validated plasma Aβ assay and is planned in follow-up work. In addition, because exosome lysates contain both membrane-associated and luminal Aβ, the present assay measures total exosome-associated Aβ and does not distinguish these pools; a proteinase K protection assay has been designed to discriminate surface-bound from luminal Aβ in follow-up work. Theoretically, in silico analyses (molecular docking and molecular dynamics simulation) can be further designed to evaluate scFv–Aβ interactions and to optimize the linker length.

## 4. Materials and Methods

### 4.1. Reagents and Materials

Hydrophilic polystyrene 96-well microtiter plates were purchased from Thermo Fisher Scientific (Waltham, MA, USA). GoldBand Plus 3-color Regular Range Protein Marker (8–180 kDa) was obtained from Yeasen Biotechnology (Shanghai, China). HRP-conjugated 6 × His Tag rabbit polyclonal antibody and HRP-conjugated goat anti-mouse IgG antibody were purchased from Jackson ImunoResearch (West Grove, PA, USA). PrimeSTAR Max Premix (2×) was acquired from Takara Bio Inc. (Dalian, China). pET-28a vector and *Escherichia coli* (*E. coli*) BL21(DE3) competent cells were purchased from TIANGEN Biotech (Beijing, China). Ni-IDA affinity chromatography column was obtained from Huiyan Bio (Wuhan, China). All other chemical reagents used were of analytical grade unless otherwise specified.

### 4.2. Preparation and Identification of Anti-Aβ mAbs

#### 4.2.1. Preparation and Purification of Anti-Aβ mAbs

BALB/c female mice (6–8 weeks old, SPF grade) were immunized with synthetic Aβ1-16, Aβ1-40, or Aβ1-42 peptides (Sangon Biotech, Shanghai, China). Splenocytes were fused with myeloma cells. Hybridomas were screened by indirect ELISA, subcloned by limiting dilution, and expanded. mAbs were purified from cell culture supernatant by protein A affinity chromatography and verified by SDS-PAGE and ELISA, using a binding buffer (20 mmol/L Na_2_HPO_4_, 150 mmol/L NaCl, pH 7.4), an elution buffer (0.1 mol/L glycine, pH 2.0) and a neutralization buffer (1 mol/L Tris-HCl, pH 8.8). The protein concentration was determined by the BCA method, and the purified mAbs were stored at −80 °C for subsequent experiments.

#### 4.2.2. SDS-PAGE and ELISA Analysis of Anti-Aβ mAbs

The purity of as-prepared mAbs was verified by 12% SDS-PAGE, followed by Coomassie brilliant blue R-250 staining. Epitope mapping and binding specificity of the mAb were determined by indirect ELISA. Briefly, 96-well ELISA plates were coated with Aβ1-16, Aβ17-26, Aβ27-40, Aβ37-42, Aβ1-40 and Aβ1-42 peptides at a concentration of 1 μg/mL (100 μL/well) in 0.05 M carbonate-bicarbonate buffer (CBS, pH 9.6) at 4 °C overnight. After washing three times with PBST (phosphate-buffered saline containing 0.05% Tween-20, pH 7.4), the plates were blocked with 10% skimmed milk in PBST at 37 °C for 2 h. Purified mAb (1 μg/mL) was added to the wells and incubated at 37 °C for 1 h, followed by three washes with PBST. HRP-conjugated goat anti-mouse IgG antibody, optimally diluted with 1% BSA-PBST (1:5000), was added (100 μL/well) and incubated at 37 °C for 30 min. For colorimetric assay, after the final wash, 100 μL of TMB chromogenic substrate was added at room temperature for 15 min, and the reaction was terminated with 100 μL of 2 M H_2_SO_4_. The absorbance at 450 nm (OD_450_) was measured using an iMark™ Microplate Absorbance Reader (Bio-Rad Laboratories, Hercules, CA, USA). Phosphate-buffered saline (PBS) was used as the negative control (NC), and all experiments were performed in triplicate.

### 4.3. Preparation and Identification of Anti-Aβ scFv

#### 4.3.1. Construction of pET-28a-scFv Recombinant Expression Vectors

The variable region gene sequences of VH and VL of the screened anti-Aβ1-16, Aβ1-40 and Aβ1-42 mAb were amplified by RT-PCR using the following primers:

VH F: cttccggaattcSARGTNMAGCTGSAGSAGTCWGG;
VH R: ggaagatctATAGACAGATGGGGGTGTCGTTTTGGC;
VL F: gggagctcGAYATTGTGMTSACMCARWCTMCA;
VL R: ggtgcatgcGGATACAGTTGGTGCAGCATC.

VH (348–351 bp) and VL (336 bp) were joined by a (Gly4Ser)3 linker (45 bp), which is widely used in scFv construction and provides sufficient length for correct VH–VL association. It gives an expected insert of 729–732 bp, while the observed 735–738 bp band corresponds to the full insert plus restriction-site/flanking sequences. A schematic of the pET-28a-scFv construct is shown in Supplementary Figure S3. The scFv gene sequences were codon-optimized for prokaryotic expression in E. coli and commercially synthesized (Sangon Biotech, Shanghai, China). The as-synthesized scFv gene and pET-28a vector were double-digested with BamHI and HindIII restriction endonucleases, and the target gene was ligated into the linearized pET-28a vector using T4 DNA ligase to construct recombinant expression vectors pET-28a-scFv1-16, pET-28a-scFv1-40 and pET-28a-scFv1-42. The recombinant vectors were transformed into E. coli DH5α competent cells, and positive clones were screened by colony PCR and verified by Sanger sequencing (Sangon Biotech, Shanghai, China).

#### 4.3.2. Expression and Purification of Anti-Aβ scFv

The sequenced pET-28a-scFv recombinant vectors were transformed into *E. coli* BL21(DE3) competent cells for induced expression. A single positive colony was picked and cultured in 5 mL Luria–Bertani (LB) medium supplemented with 100 μg/mL ampicillin (LBA) at 37 °C overnight with shaking (220 r/min). The seed culture was inoculated into 500 mL fresh LBA medium at a volume ratio of 1:100 and cultured at 37 °C with shaking (220 r/min) until the OD_600_ value reached 0.6–0.8. Isopropyl β-D-1-thiogalactopyranoside (IPTG) was added to a final concentration of 0.5 mmol/L to induce scFv expression at 30 °C for 12 h with shaking (180 r/min). The bacterial culture was harvested by centrifugation at 12,000× *g* for 5 min at 4 °C, and the bacterial pellet was resuspended in pre-cooled PBS (pH 7.4). The bacteria were lysed by intermittent ultrasonic disruption at 4 °C (20 min, 3 s on/4 s off, 105 W), and the lysate was centrifuged at 12,000× *g* for 20 min at 4 °C to collect the supernatant. The scFv protein with a 6 × His tag in the supernatant was purified by Ni-IDA affinity chromatography according to the manufacturer’s instructions. The protein concentration of the purified scFv was determined by the BCA method, and the samples were stored at −80 °C for subsequent use.

#### 4.3.3. SDS-PAGE and ELISA Analysis of Anti-Aβ scFv

The expression and purity of the purified scFv were verified by 12% SDS-PAGE and Coomassie brilliant blue R-250 staining. The binding specificity of scFv was identified by indirect ELISA. The experimental procedure was consistent with that of mAb identification, with the following modifications: purified scFv (1 μg/mL) was used as the primary antibody, and HRP-conjugated 6 × His Tag rabbit polyclonal antibody, optimally diluted with 1% BSA-PBST, was used as the secondary antibody. PBS was used as the negative control, and all experiments were performed in triplicate.

### 4.4. Construction of mAb-Based and scFv-Based CLIA

A double-antibody sandwich CLIA for the detection of Aβ1-40 and Aβ1-42 was established using mAb1-16 as the capture antibody and ALP-conjugated mAb1-40/mAb1-42 as the detection antibody. Carboxyl magnetic beads (1 μm) were coated with mAb1-16 at a concentration of 0.25 mg/mL (50 μL/test) in 1% BSA-Tris-HCl (pH 7.4) at 37 °C for 2 h and then blocked with 2% BSA-Tris-HCl for 1 h. Aβ1-40 and Aβ1-42 standard peptides were diluted to a series of concentrations (0, 1, 5, 10, 25, 50, 100, 200, 400, 800, 1200 pg/mL) with 1% BSA-Tris-HCl (pH 7.4). Eventually, 100 μL of standard peptide solution and 100 μL of optimally diluted ALP-conjugated mAb1-40/mAb1-42 were added to the magnetic bead-coated reaction cups and incubated at 37 °C for 30 min with shaking. After washing the magnetic beads three times with PBST, 200 μL of AMPPD substrate was added, and the relative light unit (RLU) was measured using an automatic chemiluminescence immunoanalyzer (Snibe Medical, Shenzhen, China). A standard curve was established with the logarithm of the standard peptide concentration as the abscissa and the logarithm of the RLU value as the ordinate, and the linear range, regression equation and limit of detection (LOD) of the assay were calculated. The LOD was defined as the concentration corresponding to the mean RLU of the blank control plus 3 times the standard deviation (S.D.).

Similarly, an scFv-based double-antibody sandwich CLIA was established using scFv1-16 as the capture reagent and ALP-conjugated scFv1-40/scFv1-42 as the detection reagent. The experimental procedures of scFv-based CLIA are identical to those of mAb-based CLIA.

### 4.5. Detection of Aβ1-40 and Aβ1-42 in Plasma Exosomes Lysates

For exosome isolation, 0.3 mL plasma was used with 75 μL extraction reagent. The particle concentration and size distribution of exosomes were characterized by NTA (Figure S1) and Western blot (Figure S2) for CD63, CD81, and TSG101, with Calnexin as a negative marker. The established mAb-based and scFv-based CLIA were used to detect the concentrations of Aβ1-40 and Aβ1-42 in the plasma exosome lysates of the AD group and the non-AD control group. Exosome lysates were diluted 1-fold in 1% BSA-Tris-HCl (pH 7.4) before CLIA. Values were within the scFv linear range (1–800 pg/mL for Aβ1-40; 1–400 pg/mL for Aβ1-42). For the mAb assay, the linear range started at 50 pg/mL; several mAb-measured values were at or below this LOD, which limits the mAb comparison. Operators were blinded to group assignment. Each sample was detected in triplicate. The Aβ1-42/Aβ1-40 ratio of each sample was calculated for comparison.

### 4.6. Statistical Analysis

All experimental data were analyzed using SPSS statistical software (IBM Corp., Armonk, NY, USA) and GraphPad Prism software (GraphPad Software, San Diego, CA, USA). Measurement data were expressed as the mean ± S.D. The normality of data distribution was assessed by the Shapiro–Wilk test, and the homogeneity of variances was assessed by Levene’s test. The independent-samples *t*-test was used for normally distributed measurements (t-values reported in Table 2), and the Mann–Whitney U non-parametric test was additionally applied to the Aβ ratio data given visible right-skewness with outliers. The receiver operating characteristic (ROC) curve was plotted to evaluate the diagnostic value of the plasma exosome Aβ1-42/Aβ1-40 ratio for AD, and the optimal cutoff value was determined according to the maximum Youden index (Youden index = sensitivity + specificity − 1). The diagnostic sensitivity, specificity, positive predictive value (PPV) and negative predictive value (NPV) of scFv-based and mAb-based assays were calculated and compared. A two-tailed *p*-value < 0.05 was considered statistically significant, and *p* < 0.01 was considered extremely statistically significant.

## 5. Conclusions

This study successfully prepared anti-Aβ scFv with high specificity and purity, and established scFv-based CLIA for the detection of Aβ1-40 and Aβ1-42 in plasma exosomes, with high sensitivity, high specificity, good precision and low cost. The detection results confirmed that the plasma exosome Aβ1-42/Aβ1-40 ratio is a potential non-invasive biomarker for differentiating clinically diagnosed AD from non-AD controls in this pilot cohort, which provides a technical approach for further evaluation in AD versus non-AD differentiation. In future research, we will expand the sample size, optimize the scFv expression system, and explore the combined detection of the plasma exosome Aβ subtype ratio with other AD biomarkers to further improve the diagnostic accuracy. In addition, a direct comparison between exosome-associated Aβ and total plasma Aβ should be considered. Since exosome lysates contain both membrane-associated and luminal Aβ, the present assay does not distinguish these pools, which is also planned in a follow-up work.

## Figures and Tables

**Table 2:** Detection of Aβ1-40 and Aβ1-42 Contents in Plasma Exosome Samples by scFv-Based and mAb-Based Assays ^1^.

Group		scFv			mAb	
	Aβ1-40 (pg/mL)	Aβ1-42 (pg/mL)	Aβ1-42/Aβ1-40	Aβ1-40 (pg/mL)	Aβ1-42 (pg/mL)	Aβ1-42/Aβ1-40
AD	49.91 ± 17.09	34.06 ± 19.81	0.70 ± 0.35	62.37 ± 18.61	51.59 ± 16.13	0.84 ± 0.19
Non-AD	35.62 ± 23.11	55.18 ± 25.93	1.82 ± 0.79	65.63 ± 19.33	92.42 ± 43.74	1.39 ± 0.46
t-value	2.222	−2.894	−5.838	−0.544	−3.917	−4.992
*p*-value	0.032	0.006	<0.001	0.589	<0.001	<0.001

^1^ Detection results represent as mean ± S.D. (n = 20).

**Table 3:** Comparison of Diagnostic Performance between scFv-Based and mAb-Based Assays ^1^.

Method	Sensitivity (%)	Specificity (%)	PPV (%)	NPV (%)	Accuracy (%)
scFv-based CLIA	90.0 (18/20)	90.0 (18/20)	90	90	90
mAb-based CLIA	85.0 (17/20)	90.0 (18/20)	89.5	85.7	87.5

^1^ PPV: positive predictive value; NPV: negative predictive value; Accuracy = (true positive + true negative)/total samples × 100%. According to the maximum Youden index (0.8), the optimal cutoff value of the Aβ1−42/Aβ1−40 ratio(scFv) for AD diagnosis was determined to be 1.075. According to the maximum Youden index (0.75), the optimal cutoff value of the Aβ1−42/Aβ1−40 ratio(mAb) for AD diagnosis was determined to be 0.924. Note: PPV and NPV were calculated under a 50% disease prevalence in this case–control study, which may not reflect values in real-world clinical populations.

## Data Availability

De-identified The original contributions presented in this study are included in the article/supplementary material. Further inquiries can be directed to the corresponding author. De-identified individual-level Aβ1-40, Aβ1-42, and Aβ1-42/Aβ1-40 ratio data are provided in Supplementary Table S4, enabling readers to verify the ROC analysis.

## Supplementary Materials

The following supporting information can be downloaded at: https://www.mdpi.com/article/10.3390/ijms272110006/s1.

## Abbreviations

The following abbreviations are used in this manuscript:

AD	Alzheimer’s disease
Aβ	β-amyloid
scFv	Single-Chain Variable Fragments
mAb	monoclonal antibody
CLIA	chemiluminescence immunoassay
CSF	cerebrospinal fluid
*E. coli*	*Escherichia coli*
CBS	carbonate-bicarbonate buffer
PBST	phosphate-buffered saline containing Tween-20
VH	variable heavy chain
VL	variable light chain
PPV	positive predictive value
NPV	negative predictive value
ROC	receiver operating characteristic
AUC	area under the ROC curve
IPTG	isopropyl β-D-1-thiogalactopyranoside
PET	positron emission tomography
RLU	relative light unit
