# Supplementary material for: Single-Chain Variable Fragments Targeting Plasma Exosome Amyloid-β Peptide Subtypes for Diagnosis of Alzheimer’s Disease
Source: Int J Mol Sci. 2026 Nov 8;27(21):10006. doi: 10.3390/ijms272110006 (PMC13763915; doi:10.3390/ijms272110006)
Supplement: Supplementary file 1 [file ijms-27-10006-s001.zip › ijms-4625735-supplementary.pdf]

Supporting Information

Single-Chain Variable Fragments Targeting Plasma Exosome Amyloid-β Peptides Subtypes for Diagnosis of Alzheimer's Disease

Xiaojin Zhou[1], Zan Wang[1] and Hui Jiang[1,*]

[1] State Key Laboratory of Digital Medical Engineering, School of Biological Science and Medical Engineering, Southeast University, Nanjing 211189, P. R. China

*E-mail: sungi@seu.edu.cn

Extraction of Plasma Exosomes

The plasma samples were centrifuged at 3000 × g for 10 min at 4°C immediately after collection to remove blood cells and cell debris, and the supernatant was collected for exosome extraction. Exosomes were extracted using a commercial exosome extraction reagent (Sangon Biotech, Shanghai, China). Firstly, 75 μL of extraction reagent was added to 300 μL of plasma supernatant, and the mixture was pipetted up and down until a flocculent colloid formed, followed by incubation at room temperature for 30 min. The mixture was centrifuged at 12000 × g for 20 min at 4°C, the supernatant was discarded, and the exosome pellet was collected. The pellet was resuspended and lysed with an equal volume of RIPA lysis buffer at room temperature for 30 min, and the lysate was stored at -80°C for subsequent detection.

Identification of Plasma Exosomes

The extracted plasma exosomes were identified by nanoparticle tracking analysis (NTA) for particle size distribution and Western blot (WB) for specific surface marker expression, which are the gold standard identification methods for exosomes recommended by the International Society for Extracellular Vesicles (ISEV). The particle size distribution, concentration and particle size homogeneity of the extracted plasma exosomes were detected using a Flow NanoAnalyzer (Nano FCM, Xiamen, China). The expression of exosome-specific surface markers (CD63, CD81) and the

negative marker (Calnexin) were detected by WB to verify the specificity of the extracted exosomes. The main experimental reagents included anti-CD63 rabbit polyclonal antibody (1:1000, Abcam), anti-CD81 rabbit polyclonal antibody (1:1000, Abcam), anti-Calnexin rabbit monoclonal antibody (1:2000, Cell Signaling Technology), HRP-conjugated goat anti-rabbit IgG secondary antibody (1:5000, Jackson ImunoResearch), and anti-TSG101 rabbit polyclonal antibody (1:1000, Abcam).

NTA results showed that the extracted plasma exosomes exhibited a typical unimodal particle size distribution in the range of 30-150 nm (**Figure S1**), which was consistent with the classic particle size characteristics of exosomes. The average particle size of the exosomes was 90.0 nm and the particle concentration of the exosome suspension was 3.95×10^{8} particles/mL. This particle concentration is relatively low for plasma-derived vesicles after polymer precipitation and is acknowledged as a limitation.

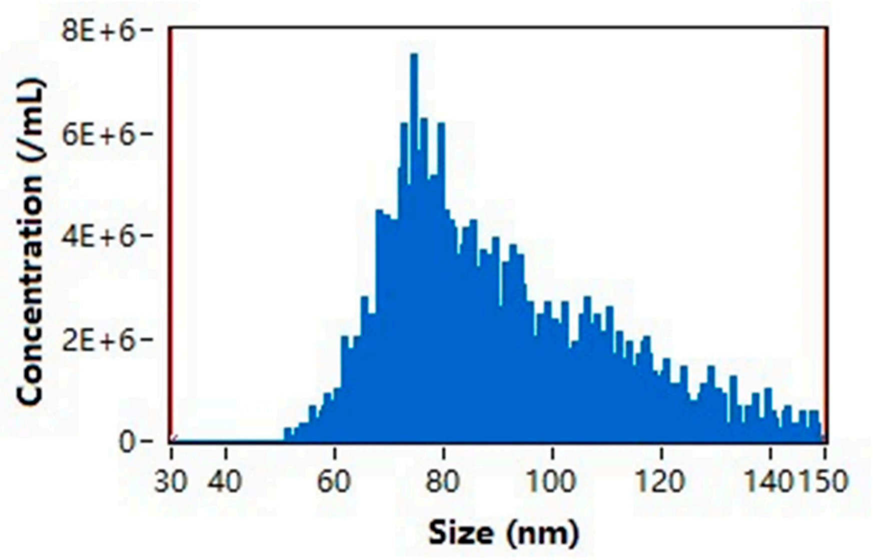

Figure S1 Nanoparticle tracking analysis (NTA) of extracted plasma exosomes.

WB results for exosome-specific surface markers and negative markers are shown in Figure S2. The extracted plasma exosome samples showed specific and clear protein bands of the exosome positive markers CD63 (~53 kDa), CD81 (~25 kDa) and TSG101 (~44 kDa) with stable expression, while the negative marker Calnexin (~90 kDa) showed no visible protein band in the exosome samples. The above results further verified the high specificity and purity of the extracted plasma exosomes, and excluded the contamination of intracellular protein impurities, ensuring the reliability of the subsequent detection results of Aβ1-40 and Aβ1-42 in plasma exosomes.

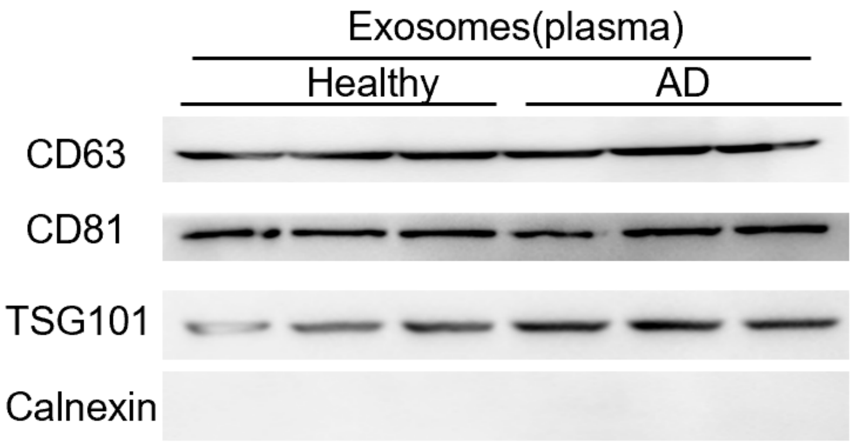

Figure S2 WB analysis of plasma exosome-specific markers.

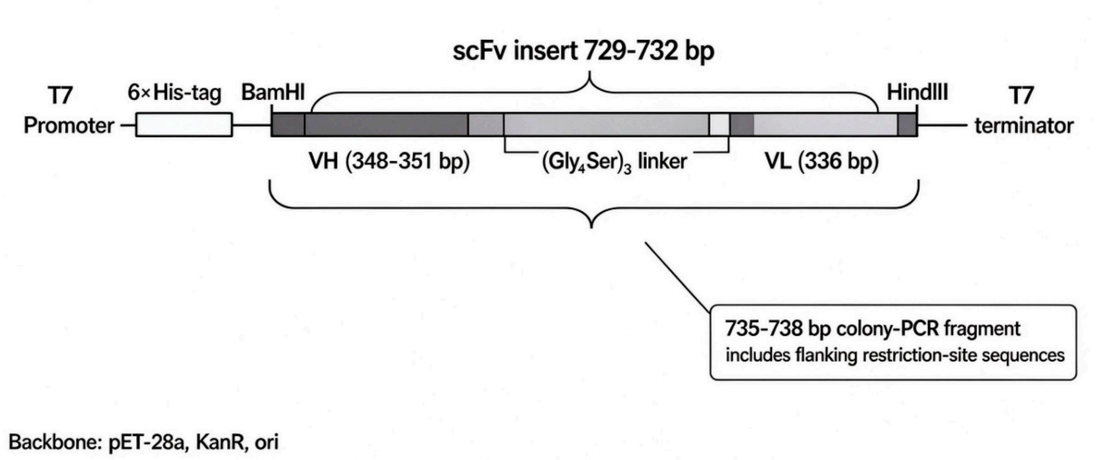

Figure S3 Schematic diagram of the construction of recombinant pET-28a-scFv.

DNA sequencing results

scFv1-16

CAGGTTACTCTGAAAGAGTCTGGCCCTGGGATATTGCAGCCCTCCCAGAC
CCTCAGTCTGACCTGTTCTTTCTCTGGGTTTTCACTGAGCACTCGTGGTATG
GGTGTGAGTTGGATTCGTCAGCCTTCAGGAAAGGGTCTGGAGTGGCTGGC
ACACATTTACTGGGATGATGACAAGCGCTATAACCCATCCCTGAAGAGCC
GGCTCACAATCTCCAGGGATGCCTCCGGAAACCAGGTATTCCTCAAGAGC
ACCAGTGTGGACACTGCAGATAGTGCCACATACTTCTGTGTTCGATACTCG
GGGGCTATGGATTATTGGGGTCCAGGAACCTCAGTCACCGTGTCCTCAGG
TGGTGGTGGTAGCGGTGGTGGTGGTAGCGGTGGTGGTGGTAGCGATGTTTT
GATGACCCAAACTCCACTCTCCCTGCCTGTCAGTCTTGGAGATCAAGCCTC
CATCTCTTGCAGATCTAGTCAGAGCATTGTACATAGTAATGGAAACACCT

ATTTAGAATGGTACCTGCAGAAACCAGGTCAGTCTCCAAAGCTCCTGATC
TACAAAGTTTCCAACCGATTTTCTGGGGTCCCAGACAGGCTCAGTGGCAG
TGGATCAGGGACAGATTTCACACTCAAGATCAGTAGAGTGGAGGCTGAGG
ATCTGGGAGTTTATTACTGCTTTCAAGTTACACATGTTCCGCTCACGTTCG
GTGCTGGGACCAAGCTGGAGCTGAAA

scFv1-40

CAGGTGCAGCTGGAGGAGTCAGGACCTGGCCTAGTGCAGCCCTCACAGAG
CCTGTCCATCACCTGCACAGTCTCTGGTTTCTCATTAACTAGCTATGGTGT
ACACTGGGTTCGCCAGTCTCCAGGAAAGGGTCTGGAGTGGCTGGGAGTGA
TATGGAGTGGCGGAAGCACAGACTATAATGCAGCTTTCATACCCAGACTG
AGCATCAGCAAGGACAATTCCAAGAGCCAAGTTTTCTTTAAAATGAACAG
TCTGCAAGCTAATGACACAGCCATATATTACTGTGCCAGAAATAGAGAGA
GGGCTATGGACTACTGGGGTCAAGGAACCTCAGTCACCGTCTCCTCAGGT
GGTGGTGGTAGCGGTGGTGGTGGTAGCGGTGGTGGTGGTAGCGATATTGT
GATGACGCAGGCTCCATCCTCCCTGGCTGTGTCAGCAGGAGAGAAGGTCA
CTATGAGCTGCAAATCCAGTCAGAGTCTGCTCAACAGTAGAACCCGAAAG
AACTACTTGGCTTGGTACCAGCAGAAACCAGGGCAGTCTCCTAAACTGCT
GATCTACTGGGCATCCACTAGGGAATCTGGGGTCCCTGATCGCTTCACAG
GCAGTGGATCTGGGACAGATTTCACTCTCACCAGCAGCAGTGTGCAGGCT
GAAGACCTGGCAGTTTATTACTGCAAGCAATCTTATAATCTGCTCACGTTC
GGTGCTGGGACCAAGCTGGAGCTGAAA

scFv1-42

CAGGTTACTCTGAAAGAGTCTGGCCCTGGGATATTGCAGCCCTCCCAGAC
CCTCAGTCTGACCTGTTCTTTCTCTGGGTTTTCACTGAGCACTCGTGGTATG
GGTGTGAGTTGGATTCGTCAGCCTTCAGGAAAGGGTCTGGAGTGGCTGGC
ACACATTTACTGGGATGATGACAAGCGCTATAACCCATCCCTGAAGAGCC
GGCTCACAATCTCCAGGGATGCCTCCGGAAACCAGGTATTCCTCAAGAGC
ACCAGTGTGGACACTGCAGATAGTGCCACATACTTCTGTGTTCGATACTCG
GGGGCTATGGATTATTGGGGTCCAGGAACCTCAGTCACCGTGTCCTCAGG
TGGTGGTGGTAGCGGTGGTGGTGGTAGCGGTGGTGGTGGTAGCGACATTG
TGATGACCCAGTCTCCATCCTCCCTGGCTGTGTCAGCAGGAGAGAAGGTC
ACTATGAGCTGCAAATCCAGTCAGAGTCTGCTCAACAGTAGAACCCGAAA

GAACTACTTGGCTTGGTACCAGCAGAAACCAGGGCAGTCTCCTAAACTGC
TGATCTACTGGGCATCCACTAGGGAATCTGGGGTCCCTGATCGCTTCACA
GGCAGTGGATCTGGGACAGATTTCACTCTCACCATCAGCAGTGTGCAGGC
TGAAGACCTGGCAGTTTATTACTGCAAGCAATCTTATAATCTGCTCACGTT
CGGTGCTGGGACCAAGCTGGAGCTGAAA

Amino acid sequences

scFv1-16

QVTLKESGPGILQPSQTLSLTCSFSGFSLSTRGMGVSWIRQPSGKGLEWLAHIY
WDDDKRYNPSLKSRLTISRDASGNQVFLKSTSVDTADSATYFCVRYSGAMD
YWGPGTSVTVSSGGGGSGGGGSGGGGSDVLMTQTPLSLPVSLGDQASISCRS
SQSIVHSNGNTYLEWYLQKPGQSPKLLIYKVSNRFSGVPDRLSGSGSGTDFTL
KISRVEAEDLGVYYCFQVTHVPLTFGAGTKLELK

scFv1-40

QVQLEESGPGLVQPSQSLSITCTVSGFSLTSYGVHWVRQSPGKGLEWLGVIWS
GGSTDYNAAFIPRLSISKDNSKSQVFFKMNSLQANDTAIYYCARNRERAMDY
WGQGTSVTVSSGGGGSGGGGSGGGGSDIVMTQAPSSLAVSAGEKVTMSCKS
SQSLLNSRTRKNYLAWYQQKPGQSPKLLIYWASTRESGVPDRFTGSGSGTDF
TLTSSSVQAEDLAVYYCKQSYNLLTFGAGTKLELK

scFv1-42

QVTLKESGPGILQPSQTLSLTCSFSGFSLSTRGMGVSWIRQPSGKGLEWLAHIY
WDDDKRYNPSLKSRLTISRDASGNQVFLKSTSVDTADSATYFCVRYSGAMD
YWGPGTSVTVSSGGGGSGGGGSGGGGSDIVMTQSPSSLAVSAGEKVTMSCK
SSQSLLNSRTRKNYLAWYQQKPGQSPKLLIYWASTRESGVPDRFTGSGSGTD
FTLTISSVQAEDLAVYYCKQSYNLLTFGAGTKLELK

Table S1 Specificity Analysis of Anti-Aβ MAb*

Antibody	Aβ1-16	Aβ17-26	Aβ27-40	Aβ37-42	Aβ1-40	Aβ1-42	NC(PBS)
mAb1-16	**2.563±0.021**	0.062±0.005	0.045±0.004	0.064±0.006	**2.762±0.032**	**2.136±0.018**	0.044±0.003
mAb1-40	0.047±0.004	0.058±0.005	**2.739±0.028**	0.057±0.005	**2.454±0.025**	0.087±0.007	0.038±0.003
mAb1-42	0.077±0.006	0.049±0.004	0.033±0.003	**2.254±0.022**	0.052±0.004	**2.567±0.026**	0.052±0.004

* OD_{450} values represent as mean ± S.D. (n=3)

Table S2 Specificity Analysis of Anti-Aβ scFv*

scFv	Aβ1-16	Aβ17-26	Aβ27-40	Aβ37-42	Aβ1-40	Aβ1-42	NC(PBS)
scFv1-16	**2.565±0.023**	0.034±0.003	0.082±0.007	0.042±0.004	**2.519±0.024**	**2.345±0.021**	0.061±0.005
scFv1-40	0.047±0.004	0.104±0.009	**2.350±0.022**	0.049±0.004	**2.199±0.020**	0.046±0.004	0.057±0.005
scFv1-42	0.054±0.004	0.038±0.003	0.057±0.005	**2.373±0.023**	0.045±0.004	**2.587±0.025**	0.059±0.005

* OD_{450} values represent as mean ± S.D. (n=3)

Table S3 RLU Values of Aβ1-40 and Aβ1-42 Detected by mAb- and scFv-based CLIA*

Concentration (pg/mL)	mAb-based CLIA (RLU)		scFv-based CLIA (RLU)	
	Aβ1-40	Aβ1-42	Aβ1-40	Aβ1-42
0	5228±105	4576±98	4876±96	5032±101
1	5276±112	4579±102	11865±215	19672±325
5	5395±121	4668±110	36370±586	60469±896
10	6129±135	5890±128	69527±1058	137038±2056
25	7536±152	5997±132	179363±2685	278237±4158
50	9659±185	11463±215	327770±4896	487177±7256
100	15190±256	20409±326	603379±8956	897363±13258
200	30838±485	36688±568	911284±13586	1362535±20458
400	58254±896	74370±1056	1483400±21956	1847075±27658
800	127008±1895	138007±2158	1837149±27586	2222999±33258
1200	216835±3256	251458±3896	2026976±30256	2416271±36158

*RLU values represent as mean ± S.D. (n=3)

Table S4 Plasma exosomal concentration of Aβ1-40 and Aβ1-42 and Aβ1-42/Aβ1-40 ratios for all samples

Sample ID	Aβ1-40 (scFv) pg/mL	Aβ1-42 (scFv) pg/mL	Aβ1-42/Aβ1-40 ratio (scFv)	Aβ1-40 (mAb) pg/mL	Aβ1-42 (mAb) pg/mL	Aβ1-42/Aβ1-40 ratio (mAb)	Diagnosis
AD1	52.15	20.24	0.388	26.83	16.76	0.625	AD
AD2	61.4	56.35	0.918	39.12	45.82	1.171	AD
AD3	42.5	29.3	0.689	64.78	57.22	0.883	AD
AD4	65	10.45	0.161	62.45	44.15	0.707	AD
AD5	28.75	15.67	0.545	73.67	65.19	0.885	AD
AD6	36.92	12.3	0.333	54.33	42.27	0.778	AD
AD7	72.5	21.64	0.298	61.93	48.79	0.788	AD
AD8	57.86	66.93	1.157	78.52	39.92	0.508	AD
AD9	81.25	35.12	0.432	63.38	49.87	0.787	AD
AD10	57.87	51.3	0.886	58.09	53.65	0.924	AD
AD11	53.32	18.11	0.34	77.26	58.09	0.752	AD
AD12	42.15	67.47	1.601	55.15	67.81	1.23	AD
AD13	38.73	36.52	0.943	63.03	77.54	1.23	AD
AD14	36.57	30.63	0.838	88.62	77	0.869	AD
AD15	54.17	35.48	0.655	92.57	69.17	0.747	AD
AD16	83.75	72.87	0.87	44.81	35.62	0.795	AD

AD17	21.23	13.37	0.63	24.62	21.75	0.883	AD
AD18	36.25	21.72	0.599	75.77	58.37	0.77	AD
AD19	32.65	19.36	0.593	84.56	56.94	0.673	AD
AD20	43.12	46.36	1.075	57.85	45.82	0.792	AD
HC1	18.44	30.16	1.636	79.36	107.82	1.359	Healthy control
HC2	12.36	24.88	2.013	76.12	104.56	1.374	Healthy control
HC3	17.67	27.26	1.543	46.43	77.67	1.673	Healthy control
HC4	45.23	57.48	1.271	79.12	84.78	1.072	Healthy control
HC5	28.39	67.45	2.376	58.63	120.35	2.053	Healthy control
HC6	28.59	49.36	1.726	68.95	102.17	1.482	Healthy control
HC7	18.37	56.04	3.051	45.26	75.05	1.658	Healthy control
HC8	59.42	60.98	1.026	92.19	114.57	1.243	Healthy control
HC9	63.77	88.64	1.39	52.83	45.07	0.853	Healthy control
HC10	23.13	84.51	3.654	77.19	82.76	1.072	Healthy control
HC11	63.67	69.36	1.089	53.81	58.32	1.084	Healthy control
HC12	13.64	26.38	1.934	20.67	21.11	1.021	Healthy control
HC13	102.53	126.71	1.236	96.35	124.18	1.289	Healthy control
HC14	32.52	82.3	2.531	86.65	221.47	2.556	Healthy control
HC15	54.83	37.67	0.687	77.59	59.67	0.769	Healthy control
HC16	20.43	36.3	1.777	43.52	48.39	1.112	Healthy control

HC17	31.76	36.3	1.143	46.63	58.56	1.256	Healthy control
HC18	40.75	49.3	1.21	78.68	79.84	1.015	Healthy control
HC19	17.53	54.92	3.133	71.77	146.51	2.041	Healthy control
HC20	19.46	37.58	1.931	60.87	115.45	1.897	Healthy control

Table S5 Precision of mAb-based and scFv-based CLIA for the detection of Aβ1-40 and Aβ1-42

Assay	Analyte	QC level	Nominal concentration (pg/mL)	Intra-assay (n = 10) Mean ± SD (pg/mL)	Intra-assay CV (%)	Inter-assay (n = 5) Mean ± SD (pg/mL)	Inter-assay CV (%)
mAb-based CLIA	Aβ1-40	Low	5	5.16 ± 0.19	3.67	4.94 ± 0.47	9.43
		Medium	100	101.29 ± 5.43	5.36	105.75 ± 6.82	6.45
		High	500	512.51 ± 28.73	5.61	487.04 ± 58.61	12.03
	Aβ1-42	Low	2	2.52 ± 0.13	5.22	2.49 ± 0.10	3.85
		Medium	50	48.77 ± 2.95	6.05	51.08 ± 3.94	7.72
		High	200	200.94 ± 13.31	6.62	207.66 ± 6.99	3.37
scFv-based CLIA	Aβ1-40	Low	5	4.95 ± 0.36	7.30	4.77 ± 0.50	10.56
		Medium	100	101.21 ± 5.55	5.48	106.36 ± 8.06	7.58
		High	500	491.05 ± 31.52	6.42	496.97 ± 58.18	11.71
	Aβ1-42	Low	2	2.47 ± 0.11	4.25	2.48 ± 0.13	5.20
		Medium	50	49.77 ± 3.54	7.11	50.23 ± 1.55	3.08
		High	200	201.33 ± 10.85	5.39	202.72 ± 25.81	12.73

Notes: Three QC levels were analyzed in ten replicates within a single run (intra-assay) and in five independent runs on five consecutive days (inter-assay). CV = (SD/mean) × 100%. Intra-assay CVs ranged 3.67–7.30% and inter-assay CVs 3.08–12.73%.
